# Management of refractory autoimmune hepatitis with rituximab: a case series

**DOI:** 10.1186/s13256-025-05595-3

**Published:** 2025-11-25

**Authors:** Nicholas M. Batt, Stephen D. Bloom, Geoffrey Haar, Amanda J. Nicoll

**Affiliations:** 1 Department of Gastroenterology, Austin Health, Heidelberg, Melbourne, VIC Australia; 2 Department of Gastroenterology, Eastern Health, Box Hill, Melbourne, VIC Australia

**Keywords:** Autoimmune hepatitis, Refractory, Rituximab, Anti-CD20, Case report

## Abstract

**Background and aims:**

Most patients with autoimmune hepatitis respond to the standard of care of prednisolone in combination with a thiopurine, or a second line of mycophenolate mofetil or tacrolimus. This study aims to add to the reported experience of using rituximab in the small numbers of patients with autoimmune hepatitis who are refractory or intolerant to these treatments.

**Case presentation:**

A retrospective single center case series was performed on six patients who were treated with rituximab for probable or definite, biopsy-proven type 1 autoimmune hepatitis over a 9-year period. They were Caucasian, three males and three females, with an age at autoimmune hepatitis diagnosis ranging from 16 to 52 years old. Three patients had cirrhosis. All six patients had trialed prednisolone, a thiopurine, and also mycophenolate mofetil prior to the rituximab. Indications for rituximab were treatment of an autoimmune hepatitis flare and potential intolerance to the second line therapy. For patients with autoimmune hepatitis flare, rituximab improved transaminases and/or total immunoglobulin G levels, lowered prednisolone dosage, and reduced the number or dose of disease-modifying agents. Four of the six cases required more than one dose of rituximab. Dosage intervals ranged from 1 month to 5 years. In one case, rituximab also effectively treated comorbid immune thrombocytopenia. No complications arose from rituximab treatment.

**Conclusion:**

Rituximab appears safe, and also effective in some patients with autoimmune hepatitis who have been refractory to standard of care. The dose reduction for other disease modifying agents makes rituximab an attractive option for this difficult to treat subset of patients.

**Supplementary Information:**

The online version contains supplementary material available at 10.1186/s13256-025-05595-3.

## Background

Autoimmune hepatitis (AIH) is an immune-mediated chronic inflammatory disorder of the liver. The exact etiology is unknown, but the onset is thought to be an immunological cell mediated process triggered by several potential factors including viral prodromes, environmental factors, and genetic predisposition. Clinically, patients often present with nonspecific symptoms such as fatigue, nausea, anorexia, and abdominal pain, though some may present with acute liver failure or asymptomatic elevated liver enzymes. Key diagnostic indicators include elevated serum aminotransferases [alanine aminotransferase (ALT), aspartate aminotransferase (AST)], hypergammaglobulinemia (especially elevated immunoglobulin G (IgG) levels), and the presence of autoantibodies such as anti-nuclear antibodies (ANA), anti-smooth muscle antibodies (SMA), and anti-liver kidney microsomal type 1 (LKM-1) antibodies [1]. A definitive diagnosis typically requires a liver biopsy, with the histological hallmark of AIH being interface hepatitis; other features include plasma cell infiltration and lobular hepatitis [2].

Standard of care treatment for AIH consists of prednisolone (PND) or other corticosteroids, in combination with a thiopurine such as azathioprine (AZA) or mercaptopurine (6MP) [1]. Second-line treatments may be used in treatment failure, incomplete response, or drug intolerance; these treatments include mycophenolate mofetil (MMF) or tacrolimus (TAC) [1, 3–5]. A small number of patients remain refractory to first-, and second-line therapies, with persistently elevated transaminases and/or total immunoglobulin G (IgG). Biologic therapy has so far been limited to patients who have failed standard second-line regimens, and has been shown to be successful in other case series [1, 4, 6, 7].

Rituximab is a chimeric monoclonal anti-CD20 antibody that can reduce B lymphocytes by targeting their cell-surface receptor, and therefore, will reduce B-cell proliferation. As the pathogenesis of AIH involves unregulated proliferation of activated plasma cells, antibody-dependent cytotoxicity of the hepatocytes, and T-cell dysregulation, rituximab is a good therapeutic option for selected patients [8]. There have been case reports of rituximab improving refractory AIH[9], overlap syndrome [10], and when used for concurrent autoimmune disease [11, 12]. Two case series have previously shown biochemical improvement in patients with AIH refractory to PND and AZA [13, 14]. A recent case series includes rituximab used as first-line therapy [7].

The aim of this case series was to add to the reported experience of using rituximab in patients with refractory AIH.

## Method

**Fig. 1:**
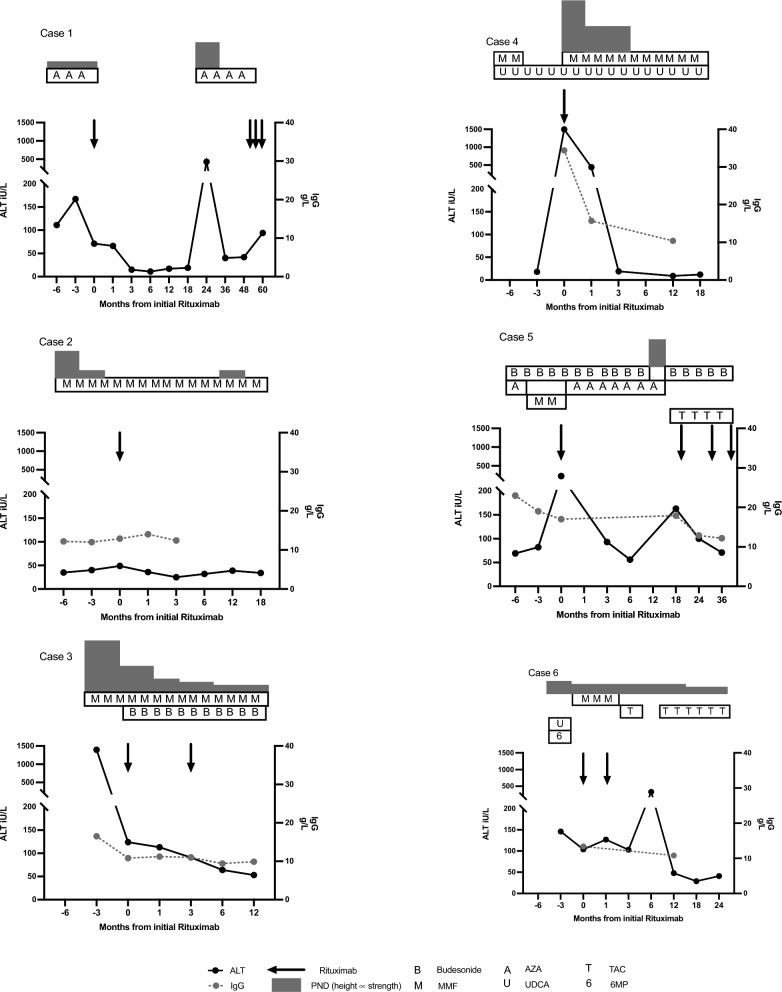
Alanine transaminase and immunoglobulin G levels of the six cases, in temporal relation to rituximab and other therapies. The height of the prednisolone box correlates to the strength of prednisolone administered. Normal range alanine transaminase 5–25 IU/ L, IgG 7–16 g/L

A retrospective single center case series was performed on patients treated with rituximab at our site between January 2013 and December 2021, with the indication being AIH. Six patients with AIH were identified who met the pretreatment diagnostic criteria for at least probable, or definite type 1 AIH according to the International Autoimmune Hepatitis Group [15]. At the time of diagnosis, liver biopsies were performed, and were consistent with AIH in all patients. All other causes of liver disease were excluded. Criteria for treatment response was as per International Autoimmune Hepatitis Group consensus; complete biochemical response (CBR) is the normalization of serum transaminases and IgG below the upper limit of normal no later than 6 months after the initiation of treatment, partial response (PR) is improvement of symptoms together with or at least 50% improvement of all liver test results during the first 2 months of treatment [16, 17]. Refractory disease was only established when compliance with medication was assured. Hepatitis B core antibody was checked prior to rituximab in all cases. Rituximab was given as a single dose infusion of 1 g; cases 1, 3, 5, and 6 did receive subsequent infusions of 1 g, as described. All received premedication with an antihistamine, corticosteroid, and paracetamol. Table 1 presents the patient characteristics of the cohort at the time of diagnosis. Figure 1 illustrates the ALT and IgG levels before and after rituximab. All patients gave written consent. The study conformed to the ethical guidelines of the 1975 Declaration of Helsinki and was approved by Eastern Health HREC (LR25/2017).

**Table 1:** Patient characteristics at the time of autoimmune hepatitis diagnosis, other autoimmune conditions, and treatments trialed prior to rituximab

Patient code	Sex	Age of diagnosis	Presentation	Cirrhosis	Other autoimmune conditions	Possible drug induced	Overlap with PBC or PSC	Simplified AIH score at presentation [18]	Pretreatment revised original score for autoimmune hepatitis (AIH) [15]	Other treatment received prior rituximab	Time from diagnosis to rituximab	Number of rituximab (dose and timing)**	Response to rituximab[16, 17]	Average daily steroid 1) 3 months pre 2) 6 months post (first rituximab) ***	Outcome (time post-first rituximab)****
1	F	16	Asymptomatic hepatitis	Yes	Pyoderma Gangrenosum	–	–	Unavailable*	Unavailable*	PND, budesonide, MMF, AZA, methotrexate	80 months	× 1 (1 g)	CBR	1) 3 mg 2) 0 mg	Alive (103 months)
2	M	49	Asymptomatic hepatitis	Yes	ITP	–	–	5	13	PND, budesonide, MMF, 6MP, UDCA	48 months	× 1 (1 g)	CBR	1) 2.5 mg 2) 0 mg	Alive (16 months)
3	F	21	Acute symptomatic hepatitis	No	–	–	–	7	16	PND, budesonide, MMF,	6 months	× 2 (1 g; 3 months)	CBR	1) 25 mg + 9 mg budesonide 2) 8 mg + 9 mg budesonide	Alive (12 months)
4	M	16	Acute symptomatic hepatitis	No	SLE	Hydroxychloroquine	PBC	7	18	PND, budesonide, MMF, UDCA	22 months	× 1 (750 mg)	CBR	1) 0 mg 2) 25 mg	Died (34 months)
5	M	22	Acute symptomatic hepatitis	No	UC	–	PSC	5	14	PND, budesonide, MMF, AZA,	32 months	× 4 (1 g; 8, 15, and 7 months)	PR	1) 0 mg + 9 mg budesonide 2) 0 mg + 9 mg budesonide	Alive (36 months)
6	F	52	Acute hepatitis	Yes	–	Amitriptyline or fluconazole	–	5	13	PND, MMF, 6MP, UDCA	67 months	× 2 (1 g; 1 month)	NR	1) 9 mg 2) 7.5 mg	Died (34 months)

The simplified AIH score should be interpreted as 1–5 = possible AIH, 6–8 = probable AIH [18]. *unavailable as archived by another institution. ** Prior to CBR if achieved, otherwise total. *** Prednisolone equivalent however unable to calculate for budesonide ****No patient received a liver transplant. *UC* ulcerative colitis, *SLE* systemic lupus erythematosus, *ITP* immune thrombocytopenia, *PSC* primary sclerosing cholangitis, *PBC* primary biliary cholangitis, *CBR* complete biochemical response, *PR* partial response *NR* No response

## Case presentation

### Case 1

A 16-year-old Caucasian female was diagnosed with AIH in 2007 via histology, biochemistry, and autoantibody tests at a pediatric hospital (Supplementary Table 1). There was a good initial response to standard therapy, with remission on PND 3 mg and AZA 150 mg daily. Shortly after her AIH diagnosis, she developed pyoderma granulosum and a vasculitic rash, which improved with brief courses of high dose PND. In 2012, an acute flare of AIH occurred on full dose AZA 150 mg daily and PND 5 mg. Cirrhosis without portal hypertension was noted through radiological imaging at this time. Multiple second-line agents were attempted but were not tolerated, including: methotrexate (acute hepatitis), MMF (severe mood swings), and budesonide (depression). She received a single 1 g dose of rituximab in 2013, which resulted in ALT normalization for 2 years on no medication. A relapse of AIH in 2015 (ALT > 600 IU/L), responded to PND 20 mg daily and reinitiation of AZA 150 mg daily. In 2018, infliximab (three doses given monthly at 5 mg/kg) was given for recurrence of pyoderma gangrenosum, which was followed by a third relapse of AIH. This relapse required a second dose of rituximab. An ALT normalization of 18 months was achieved. Further single doses of rituximab were given in November 2021, and January 2022 for failure to improve on AZA monotherapy due to intolerance of PND. She has had four single doses of 1 g rituximab in total, with no complications attributable to the medication and prolonged biochemical response.

### Case 2

A 49-year-old Caucasian man was diagnosed with seronegative AIH and Child Pugh A cirrhosis in 2017 after presenting with painless jaundice. Liver histology was compatible with AIH and cirrhosis, and the total IgG was raised (19.8 g/L). A simultaneous diagnosis of immune thrombocytopenia (ITP) was made, and a complete response to both was seen with PND. He was commenced on 75 mg 6MP as a PND sparing therapy, but this was ceased due to ageusia. Budesonide was poorly tolerated with psychological side effects. Complete biochemical response was ultimately achieved with MMF 1 g BD and low dose PND. Episodic high dose PND courses were only required for ITP. Owing to MMF side effects at higher dosing to control AIH and ITP, a single rituximab 1 g dose was given 48 months after diagnosis along with a prednisolone wean, with sustained complete biochemical response and platelet count improvement. The AIH has remained in remission on MMF 1 g BD (40 months).

### Case 3

A 21-year-old Caucasian man presented with jaundice and was diagnosed with AIH in 2017, with a bilirubin 155 μmol/L and ALT 3087 IU/L. Liver histology was consistent with AIH, antinuclear antibodies (ANA) were positive (1:160 speckled) and total IgG was 22.4 g/L. PND 60 mg daily was commenced, with relapse when reduced to 30 mg daily. Management was changed to budesonide 9 mg daily along with MMF 1 g BD, with cessation of PND. Rituximab 1 g was administered at 6 months after the initial AIH diagnosis, due to nonresponse to MMF and budesonide, and a good partial response was seen. A second rituximab 1 g dose was given 3 months later along with increased dose of MMF 2 g BD, which produced a complete biochemical response, allowing the reduction of PND to 3 mg daily 18 months postdiagnosis.

### Case 4

A 16-year-old Caucasian man was diagnosed with AIH in 2017 having presented with fatigue, fevers, rash, cervical lymphadenopathy, and raised transaminases. He had consistent liver histology, positive ANA (> 1:2560), positive anti-smooth muscle antibodies (SMA) (1:160), and elevated IgG (35.9 g/L). Initial therapy with PND 40 mg daily was complicated by steroid induced anxiety, necessitating a change to budesonide 9 mg daily. Hydroxychloroquine 200 mg BD was also initiated due to concurrent systemic lupus erythematosus (SLE), meeting European Alliance of Associations for Rheumatology (EULAR)/American College of Rheumatology (ACR) criteria[19]. The response to budesonide was suboptimal; therefore, combination PND and MMF 500 mg BD was introduced at 2 months after diagnosis, with good partial response. This was used instead of a thiopurine owing to evidence that it may be more effective in first-line therapy. Dose escalation of MMF higher than 500 mg BD was limited by neutropenia. PND was ceased after 6 months due to significant side effects, including insomnia, anorexia, and self-harm ideation. AIH/primary biliary cholangitis (PBC) variant with an elevated alkaline phosphatase (ALP) and gamma glutamyltransferase (GGT) was diagnosed in June 2018, on the basis of the cholestatic biochemistry and review of the histology which showed ductular inflammation [20]. AMA was negative at diagnosis and was not repeated. Ursodeoxycholic acid (UDCA) 250 mg BD was commenced which improved ALP, GGT as well as ALT. MMF and hydroxychloroquine were withheld for surgical management of a fracture and the AIH relapsed in August 2019. Rituximab 750 mg (375 mg/m^2^) single dose was given, at the same time MMF 1 g BD and PND 50 mg daily were recommenced with complete biochemical response achieved. He was lost to follow up from August 2021. He died of suicide in July 2022.

### Case 5

A 23-year-old Caucasian man was diagnosed with synchronous AIH/primary sclerosing cholangitis (PSC) variant in 2016, with positive ANA (1:320), positive pANCA, mildly positive SMA (1:20) and an elevated IgG (23 g/L). The liver biopsy was consistent with AIH but with a marked ductular reaction resulting in a small duct PSC diagnosis. The magnetic resonance cholangiopancreatography (MRCP) was normal. He had a background of ulcerative colitis diagnosed also in 2016, which remained quiescent on mesalazine 1.2 g BD. He had a partial response to PND at 4 weeks, and was then changed and maintained on budesonide 9 mg and AZA 150 mg daily. A mild relapse in 2019 (ALT 83 IU/L) resulted in a trial of MMF but with no improvement, a single dose of rituximab 1 g was given in June 2019 and AZA 150 mg daily was restarted. He has had three more doses of rituximab 1 g (February 2021, May 2022, and December 2022), along with TAC initiation, with failure to achieve complete biochemical response but significant improvement in liver biochemistry following each rituximab.

### Case 6

A 58-year-old Caucasian woman had seronegative but biopsy-proven AIH diagnosed in 2012. She remained on PND 10 mg, and 6MP, but had been lost to follow up. She presented with a relapse in 2018 with ALT 390 IU/L, IgG 19 g/L, and a biopsy showing signs of active AIH, and cirrhosis (Child–Pugh B9, bilirubin 47 µ/L, albumin 25 g/L, mild ascites). Second-line treatment with MMF and UDCA did not induce remission and was poorly tolerated. Rituximab 1 g was given May 2018, with a second dose 1 month later, with nonresponse to the both doses. She had a neutropenia post the initial rituximab infusion (nadir of 0.32 × 10^9^/mL) but not after the second infusion. She was maintained on PND 7.5 mg, however relapsed 6 months later (ALT 545 iU/L). TAC 1 mg daily was introduced with good partial response. She subsequently had multiple admissions for decompensated liver disease, which was driven by poor medication compliance. She died due to an upper gastrointestinal hemorrhage from esophageal varices in 2021.

## Discussion

This retrospective single center case series of six patients shows that rituximab is effective for some patients with AIH refractory to standard of care or with drug intolerance (Table 1). Prednisolone dosage and the number or dose of disease-modifying agents was reduced after rituximab (Fig. 1, Table 1). In case 1, the patient even successfully remained off treatment for 2 years. No patient received a liver transplant. All cases received other therapies around the time of rituximab administration, which needs to be considered when interpreting the data, along with the heterogeneity of the population.

Rituximab blocking of the CD20 cell-surface receptor has been shown to generally effectively suppress inflammation in autoimmune disease [21]. Case 2 shows that rituximab can be used to treat comorbid ITP as well as AIH, which is comparable with the outcomes of another case report [11]. Rituximab has also been shown to improve ALP and IgM in patients with PBC with an incomplete response to UDCA [22].

No severe complications occurred due to rituximab for AIH treatment. Infusion reactions are the most common side effect with rituximab but did not occur in this series [23]. There is potential for serious infections with rituximab, especially with severe hypogammaglobulinemia, but no infections occurred in our series [24]. Late onset neutropenia with rituximab has been documented after its use in stem-cell transplantation and in rheumatic diseases, and was briefly seen in case 6 with Child Pugh B cirrhosis [25].

Rituximab was used as rescue therapy in these cases with the planned strategy of transitioning to a new second-line agent. However, four of the six cases required more than one dose; the time between the second dose ranged from 1 month (case 6) to 5 years (case 1). The ideal dosing and regimen for rituximab in AIH is not clear, but some authors recommend ongoing therapy. The protocol for rheumatoid arthritis of induction on day 1 and 15, with subsequent courses administered every 24 weeks, may be applicable to refractory patients with AIH [23]. Two other large case series have shown similar results to ours [7, 14]. Our cohort adds another six cases of difficult to treat AIH to the 63 in the literature [7, 13, 14].

This case series shows that rituximab can be considered for patients with autoimmune hepatitis (AIH) who are refractory to or intolerant of conventional first- and second-line immunosuppressive therapies (such as prednisolone, thiopurines, mycophenolate mofetil). It is also an option for patients with coexisting autoimmune conditions, including ITP, that could also benefit from rituximab, or when despite steroid sparing agents, long-term corticosteroid is still required to prevent toxicities. Further investigation is necessary to identify the patient population most likely to respond favorably.

The ideal dose of rituximab for AIH refractory to other therapies is not well elucidated. In the patients presented here, the dose was determined by the treating clinician depending on the response to the first dose. In cases that responded well to the first dose, only a single dose was given (cases 1, 2, and 4). Case 3 had a CBR after the second dose, given after a PR to the first dose. Case 5 received four doses and achieved a good PR, but interpretation was difficult owing to the presence of PSC overlap. Case 6 was already cirrhotic owing to prior noncompliance at the time of presentation to our unit, and only had a minor response to two doses of rituximab and it was felt that further dosing was unlikely to help.

Rituximab’s use has important contraindications and considerations. Absolute contraindications include known hypersensitivity to rituximab, active severe infections, and active hepatitis B virus infection (requiring screening and prophylaxis if present). Caution is advised in patients with significant pre-existing cardiac or pulmonary conditions, or severe hypogammaglobulinemia (due to increased infection risk). In addition, live vaccines should be avoided during and for a period following rituximab treatment.

## Conclusion

Rituximab appears effective in some instances and appears safe in AIH patients refractory to standard of care treatment, but further evaluation is required and the ideal dosing regimen is yet to be confirmed. The significant reduction in dosage of other medications that can be achieved in this difficult to treat group makes this an attractive option in a subset of patients, helped by its reduced cost with the availability of biosimilars.

## Supplementary Information

Additional file 1.

## Data Availability

All relevant data is included in the manuscript.

## Abbreviations

AIH: Autoimmune hepatitis
PND: Prednisolone
AZA: Azathioprine
6MP: Mercaptopurine
MMF: Mycophenolate mofetil
TAC: Tacrolimus
UDCA: Ursodeoxycholic acid
IBD: Inflammatory bowel disease
ITP: Immune thrombocytopenia
PBC: Primary biliary cholangitis
PSC: Primary sclerosing cholangitis
SLE: Systemic lupus erythematosus
ALT: Alanine aminotransferase
AST: Aspartate aminotransferase
GGT: Gamma-glutamyl transferase
ALP: Alkaline phosphatase
IgG: Immunoglobulin G
ANA: Antinuclear antibody
SMA: Anti-smooth muscle antibody
LKM-1: Anti-liver-kidney microsomal antibody
SLA: Soluble liver antibody
AMA: Anti-mitochondrial antibody
pANCA: Perinuclear anti-neutrophil cytoplasmic antibody
