# Supplementary material for: Management of refractory autoimmune hepatitis with rituximab: a case series
Source: J Med Case Rep. 2025 Nov 25;19:614. doi: 10.1186/s13256-025-05595-3 (PMC12645696; doi:10.1186/s13256-025-05595-3)
Supplement: Supplementary file 1 — Additional file 1. [file 13256_2025_5595_MOESM1_ESM.docx]

**SUPPLEMENTARY MATERIAL**

Bloods and histology at time of Autoimmune Hepatitis diagnosis

|  | Lab values | | | | | | | | | | | | | | | | | | | Liver histology | | | | | |
| --- | --- | --- | --- | --- | --- | --- | --- | --- | --- | --- | --- | --- | --- | --- | --- | --- | --- | --- | --- | --- | --- | --- | --- | --- | --- |
| Patient code | ALT | AST | ALP | GGT | Bili | Albumin | Hb | WCC | Neut | Plt | INR | Cr | ANA | SMA | LKM-1 | AMA | IgG | Seropositive other defined autoantibodies | HLA DR3 or DR4 | Interface hepatitis | Predominantly lymphoplasmacytic infiltrate | Rosetting of liver cells | None of the above | Biliary changes | Other changes |
| 1 | UKN | UKN | UKN | UKN | UKN | UKN | UKN | UKN | UKN | UKN | UKN | UKN | UKN | 1:640 | <1:10 | <1:10 | UKN | UKN | UKN | 1 | 1 | 1 | 0 | 0 | 0 |
| 2 | 748 | 940 | 181 | 357 | 43 | 33 | 162 | 6.7 | 4.13 | 75 | 1.3 | 81 | <1:100 | <1:10 | <1:10 | <1:10 | 19.8 | - | DR4 | 1 | 0 | 0 | 0 | 1 | 0 |
| 3 | 3087 | 2227 | 143 | 89 | 155 | 35 | 135 | 6.5 | 2.84 | 215 | 1.2 | 68 | 1:160 | <1:10 | <1:10 | <1:10 | 22.4 | - |  | 1 | 1 | 0 | 0 | 0 | 0 |
| 4 | 845 | 1095 | 138 | 143 | 46 | 35 | 124 | 4.1 | 1.3 | 162 | 1.5 | 60 | >1:2560 | 1:160 | <1:10 | <1:10 | 35.9 | - |  | 1 | 1 | 0 | 0 | 0 | 0 |
| 5 | 296 | 148 | 532 | 287 | 13 | 36 | 145 | 5.5 | 2.9 | 256 | 1.1 | 128 | 1:320 | 1:20 | <1:10 | <1:10 | 23 | pANCA |  | 0 | 1 | 0 | 0 | 1 | 0 |
| 6 | 895 | 461 | 114 | 66 | 7 | 37 | 137 | 4.5 | 2.7 | 232 | 1 | 59 | <1:80 | <1:40 | <1:10 | <1:10 | 19 | - |  | 1 | 0 | 0 | 0 | 0 | 0 |

**Supplementary Table 1.** Bloods and histology at time of Autoimmune Hepatitis diagnosis. Other defined autoantibodies include pANCA, anti-LCI, anti-ASGPR, anti-LP. UKN (unknown), ND (not detected). Normal range ALT 5-35 U/L, AST 5-30 U/L, ALP 30-110 U/L, GGT 5-35 U/L, Bili <21 micromol/L, Albumin 35-52 g/L, Hb 115-155 g/L, WCC 4-12 x10^9^/L, Neut 2-8 x10^9^/L, Plt 150-400 x10^9^/L, Cr 45-90 micromol/L, IgG 6.1-16.2 g/L
